# Febrile seizure in children with COVID-19 during the Omicron wave

**DOI:** 10.3389/fped.2023.1197156

**Published:** 2023-10-20

**Authors:** Pu Xu, Xuelian Chen, Jianguo Zhou, Wenhao Zhou, Laishuan Wang

**Affiliations:** ^1^ National Health Commission Key Laboratory of Neonatal Diseases, Department of Neonatology, Children’s Hospital of Fudan University, Shanghai, China; ^2^ Department of Obstetrics and Gynecology, Renji Hospital of Shanghai Jiao Tong University School of Medicine, Shanghai, China

**Keywords:** febrile seizures, COVID-19, Omicron, children, febrile convulsions

## Abstract

**Objective:**

To explore the clinical characteristics and prognosis of febrile seizure in children with COVID-19.

**Methods:**

This study is a single-center retrospective cohort study. The cases included febrile seizures in children with COVID-19 admitted to the Renji Hospital from April 7th, 2022 to June 2nd, 2022. We compared children with and without febrile seizures in their clinical characteristics such as sex, age, symptoms, seizure manifestation, COVID-19 severity, and SARS-CoV-2 nucleic acid test results. The children with febrile seizures were followed up by telephone and outpatient service about one month after the nucleic acid turned negative and discharged from the hospital.

**Results:**

A total of 585 cases of children with COVID-19 were included in the analysis. There were 15 children (1.8%) with febrile seizures, age from six months to three years old, nine boys (60.0%) and six girls (40.0%). The manifestations of febrile seizures were all generalized tonic-clonic seizures. The median nucleic acid negative conversion time was 11 (IQR:10.75,13) days. Our first comparison involved comparing children without underlying diseases; there was no significant difference in sex, COVID-19 severity, and clinical manifestations, but there was an age difference (2 vs. 1.3, *P* = 0.047). There was no difference in SARS-CoV-2 nucleic acid negative time between the two groups (11d vs. 13d, *P* = 0.128). One child had new clinical manifestations during the follow-up, but his EEG and MRI were normal.

**Conclusion:**

Febrile seizure may be children's primary neurological manifestation of COVID-19. It may occur in children with no history of epilepsy and is not associated with severe illness. The long-term neurological outcomes of these children should be followed up.

## 1. Introduction

Children with COVID-19 account for about 10% of all COVID-19 cases and have a less severe illness compared with adult patients (1). The most common symptoms are fever and cough and other symptoms include fatigue, myalgia, nausea and vomiting, abdominal pain, and diarrhea. Most of the symptoms resolve within one week (2, 3). Neurological symptoms varied between new onset seizures, anosmia, ageusia and focal arteriopathy in many studies (4–6). During the Omicron strain epidemic, the number of COVID-19 infections, hospitalization and neurological symptoms were higher than other SARS-CoV-2 variant outbreaks (7, 8). Encephalitis and death have been reported in COVID-19 infected children in Hong Kong, Japan, Taiwan and other regions (9). Seizure in children with COVID-19 may be caused by viral encephalitis and brain injury.

This study evaluated 585 cases of 0–3 year old young children who were hospitalized for symptomatic COVID-19 infection during the Omicron strain epidemic in Shanghai, China. We identified 15 children who developed febrile and summarized their clinical characteristics in order to provide a reference for the diagnosis and treatment of seizure in children with COVID-19.

## 2. Method

### 2.1. Study design and participants

This single-center retrospective study was conducted at Renji Hospital (South branch), School of Medicine, Shanghai Jiao Tong University, from April 7, 2022, to June 2, 2022. The hospital was designated treatment of children with COVID-19 infection during the COVID-19 pandemic in Shanghai, China. This study was approved by the ethics committee of the Children's Hospital of Fudan University (IRB No. 2022–82).

Children of age 0–3 years who had symptomatic COVID-19 infection and without underlying chronic diseases were included in the study. The study patients were divided into two groups: COVID-19 children who had febrile seizures and COVID-19 children who did not have febrile seizure during the course of infection. The disease severity of COVID-19 infection children was classified based on the WHO COVID-19 Clinical Progression Scale (9), including five categories: uninfected, mild, moderate, severe, or death.

### 2.2. Data collection

Demographic and clinical data of including gender, age, PCR test, infection severity, vaccination status (no data in Table 1), and nucleic acid test negative conversion time, were collected by reviewing the hospital electronic medical records.

**Table 1:** General data, clinical manifestations and telephone follow-up of children with febrile seizure.

	Gender	Age	Severity of illness	Nucleic acid conversion time (day)	Past history	Vaccination	COVID-19 related symptoms	Convulsive form	Telephone follow-up
1	Male	1 year 6 months	Mild	14	N	N	Fever	Fever during seizures, 1 general seizure lasting 1–2 min	Normal
2	Male	2 years	Mild	11	There was one febrile seizure at the age of 1 years and 1 years 10 months.	N	Fever, Fatigue	Fever during seizures, 1 general seizure lasting 1–2 min	Normal
3	Female	1 year 3 months	Mild	13	N	N	Fever, Cough, Fatigue	Fever during seizures, 1 general seizure lasting 1–2 min	Normal
4	Male	3 years	Moderate	9	N	N	Fever	Fever during seizures, 1 general seizure lasting 1–2 min	Normal
5	Male	3 years	Moderate	10	N	N	Fever	Fever during seizures, 1 general seizure lasting 1–2 min	Normal
6	Male	8 months	Moderate	15	N	N	Fever, Cough	Fever during seizures, 1 general seizure lasting 1–2 min	Normal
7	Female	3 years	Moderate	8	N	N	Fever, Cough, Diarrhea	Fever during seizures, 1 general seizure lasting 1–2 min	Normal
8	Male	2 years	Moderate	13	N	N	Fever	Fever during seizures, 1 general seizure lasting 1–2 min	Normal
9	Male	5 months	Moderate	11	N	N	Fever, Cough	Fever during seizures, 1 general seizure lasting 1–2 min	Normal
10	Male	1 years 4 months	Moderate	12	N	N	Fever	Fever during seizures, 1 general seizure lasting 1–2 min	There is a significant increase in night terrors/nocturnal crying.
11	Female	2 years	Mild	11	N	N	Fever, Cough	Fever during seizures, 1 general seizure lasting 1–2 min	Normal
12	Male	2 years 8 months	Mild	11	N	N	Fever, Cough	Fever during seizures, 1 general seizure lasting 1–2 min	Normal
13	Female	1 years 10 months	Mild	15	N	N	Fever, Cough	Fever during seizures, 1 general seizure lasting 1–2 min	Normal
14	Female	2 years 3 months	Mild	9	N	N	Fever, Fatigue	Fever during seizures, 1 general seizure lasting 1–2 min	Normal
15	Female	2 years 5 months	Moderate	12	N	N	Fever	Fever during seizures, 1 general seizure lasting 1–2 min	Normal

### 2.3. Laboratory analysis

All cases were laboratory-confirmed as infected with the Omicron variant of SARS-COV-2 by reverse transcriptase-polymerase chain reaction (RT-PCR) nucleic acid test. Nasal swabs were conducted by trained nurses using a standard procedure. Specimens underwent RT-PCR tests for SARS-CoV-2 nucleocapsid gene targets with standardized methods and interpretive criteria. Two SARS-CoV-2 genes, including ORF1ab, and N were detected using SARS-CoV-2 nucleic acid detection, with a cycle threshold of <35 as a positive result, following the manufacturer's instructions.

### 2.4. The definition of nucleic acid conversion

The nucleic acid conversion was defined as two consecutive daily negative SARS-CoV2 PCR results. Nucleic acid conversion time was defined as the time duration from the onset of symptoms to the first time of the two (first negative day) consecutive negative PCR tests.

### 2.5. Follow-up

The cutoff point of follow-up was about one month (4–5 weeks) after negative PCR test and the patient was discharged from the hospital. Telephone follow-up: whether there were still COVID-19-related clinical manifestations after discharge, the time and duration of these symptoms, and whether appetite, body weight, sleep and energy changed after discharge.

### 2.6. Statistical analysis

The study used Microsoft Access 13.0 to create the database and SPSS 20.0 for statistical analysis. The inter-group comparisons of clinical characteristics in the seizure and the non-seizure groups were conducted by chi-square and rank-sum tests.

## 3. Results

During the study period, a total of 871 children were admitted to Renji Hospital for treatment of symptomatic COVID-19. Of these, 585 children age 0–3 years who did not have underlying diseases were included in the study. Patient demographic and clinical presentations are summarized in Table 1.

### 3.1. Clinical presentations of children with seizure during COVID-19 infection

**Figure 1:**
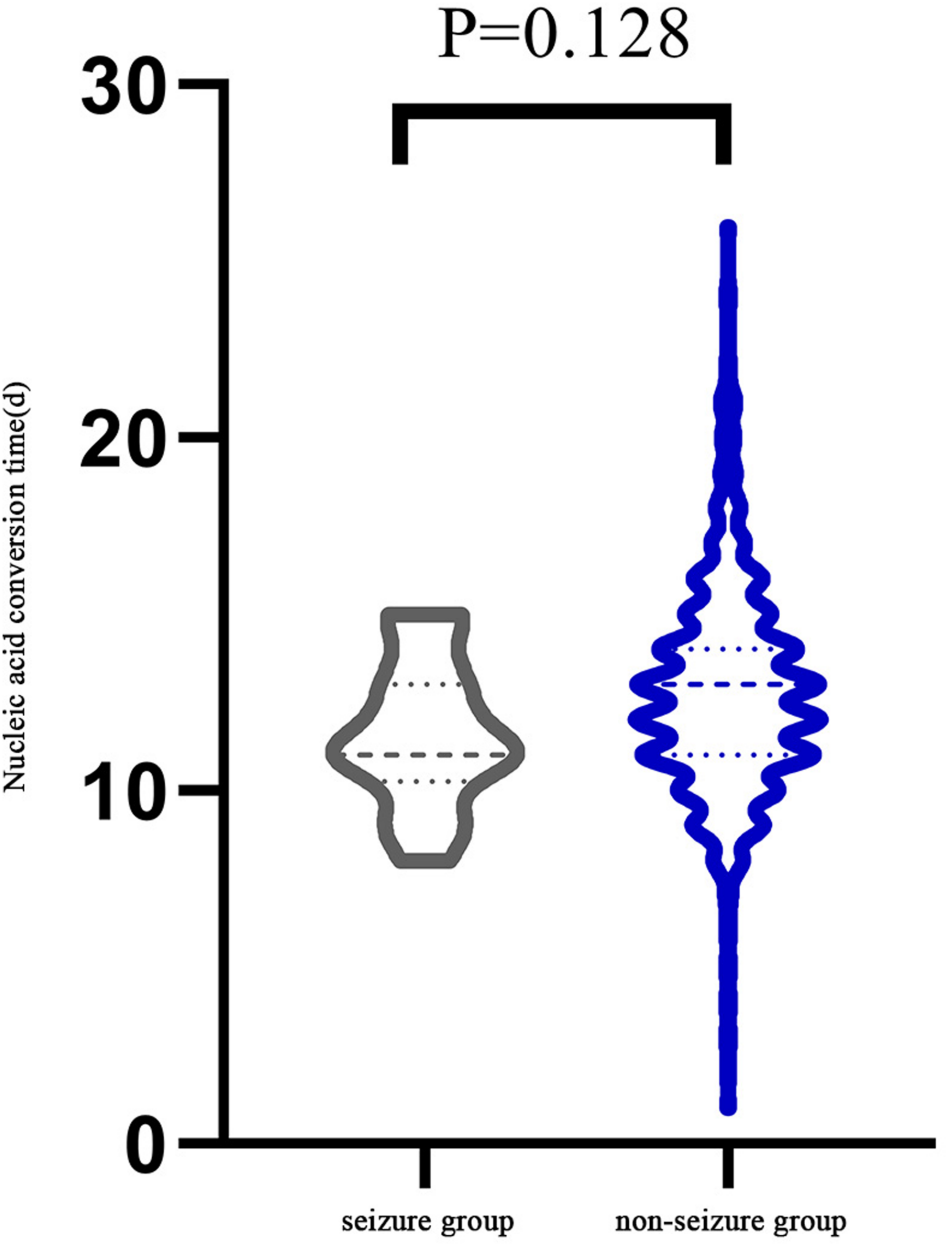
Comparison of the time required for each group since the onset of symptoms or the first positive nucleic acid.

There were 15 children, 9 boys and 6 girls, had febrile seizure during the course of infection (Table 1). They were 6 months to 3 years old, with a median age of 2 years (IQR:1.3,2.7). Seven (47%) had mild infection and eight (53%) had moderate infection. All 15 children had fever during the infection. The fevers lasted 2–4 days with peak temperatures of 38–40°C. Other symptoms included cough (*n* = 9, 60%), runny nose (*n* = 4, 27%), fatigue (*n* = 3, 20%), vomiting (*n* = 1, 7%), no diarrhea and other infection in the course of infection. All 15 children had 1–2 min generalized tonic-clonic seizures while febrile. All of them had only one seizure episode and none of them were treated antiepileptic medication. The median time of nucleic acid negative conversion in the 15 children was 11 (IQR:10.75, 13) days (Figure 1).

### 3.2. Comparison between children with and without febrile seizure during COVID-19 infection

Demographics and clinical presentations of children in the seizure group and non-seizure group were compared (Table 2). There was no significant difference in sex, number of patients in different categories of infection severity and clinical manifestation between the two groups. However, there was a significant difference in median age (2 vs. 1.3 years, *P* = 0.047). There was no significant difference in nucleic acid negative conversion time between the two groups (11d vs. 13d, *P* = 0.128) (Figure 1).

**Table 2:** Comparison of clinical characteristics between febrile seizure group and non-febrile seizure group.

	Febrile seizure group (*N *= 15)	Non-febrile seizure group (*N* = 570)	*P*
Gender (%)			
Male	9 (60.0%)	328 (57.5%)	0.692
Female	6 (40.0%)	242 (42.5%)	0.692
Median age (IQR, year)	2 (1.3, 2.7)	1.3 (0.75, 2)	0.047
Severity of illness (%)			
Mild	7 (46.7%)	353 (61.9%)	0.141
Moderate	8 (53.3%)	217 (38.1%)	0.141
Severe	0 (0%)	0 (0%)	1
General symptoms (%)			
Fever	15 (100%)	540 (94.7%)	1
Cough	8 (53.3%)	319 (56.0%)	1
Diarrhea	0 (0%)	77 (13.5%)	0.248
Vomit	1 (6.7%)	61 (10.7%)	1
Fatigue	3 (20.0%)	96 (16.8%)	0.741

### 3.3. Follow-up

The parents of 15 children with febrile seizures were followed up by telephone at about one month (4–5 weeks) after negative PCR test. Fourteen children (93%) were reported healthy with no persistent or new COVID symptoms or other health problems after discharge. During the telephone follow-up, the parents of Child #10 (in Table 1) reported that the child had significant increase in numbers of night terrors/crying at night but no seizures, fever, cough, gastrointestinal abnormalities and other symptoms. Two months after discharge, this child was readmitted to our hospital for neurological evaluation. No abnormalities were seen on the video EEG and brain MRI.

## 4. Discussion

Febrile seizure is one of the most common neurological disease in infants and young children, which usually occurs from 6 months to 5 years old, and the incidence rate is 2% and 4% in children under five years old. The prognosis is usually good, although about 1/3 of children are at risk of recurrence (10). The pathogenesis of febrile seizure is not clear and is generally believed to be caused by multiple factors, including, but not limited to, elevated body temperature, viral infection, some vaccinations, family inheritance, etc. al (11). Viral infections, especially those that cause high fever, have been shown to increase neuronal excitability and lower seizure threshold, especially in the immature nervous system (12). Common viruses that cause febrile seizure include human herpesvirus 6, influenza, adenovirus, parainfluenza and chickenpox (13).

We here report 15 cases of novel coronavirus Omicron variant infection with febrile seizure in children, accounting for 1.8% of the children's cases treated in designated hospitals. A US study showed that 0.5% of children with COVID-19 were diagnosed with febrile seizure, and most of them had no co-infection, with about 9 percent of them requiring intensive care (14). In an Italian multicenter study, Garazzino et al. evaluated 168 children with COVID-19 and reported the prevalence of afebrile and febrile seizures to be 1.8% and 1.2% (3). During 2019.01–2020.12, there were 29,825 cases of febrile children without COVID-19 treated in Shanghai with a sex ratio of 1.4 (17,377 males and 12,448 females). The age ranged from 5 months to 12 years old, with an average of (5.02 ± 1.64) years. Febrile seizure occurred in 252 cases, and the incidence of febrile seizure was 0.84%. We compared our febrile seizure group with children with febrile seizure without COVID-19 in Shanghai, there were no significant differences in incidence (1.8% vs. 1.2%, *P* = 0.095) and gender (*P* = 0.783) between the two age-matched groups (15). These findings suggest that febrile seizure is not a common neurological manifestation of COVID-19 infection (14). In another study, the number of children admitted to emergency departments for febrile seizure was significantly lower than in previous years due to the habit of wearing masks and social isolation during the COVID-19 epidemic (16).

The age range of 15 coronavirus Omicron variant infection children were from 6 months to 3 years old, which conformed to the typical age range of febrile seizure, and all the seizure were generalized (tonic-clonic) seizure. Fifteen children had only one seizure in the course of the disease, and the duration was 1–2 min, which was consistent with simple febrile seizure.

Some cases have been reported in South Africa and Sweden in children outside the typical age range for febrile seizure (17, 18).

Available evidence indicates that novel coronavirus is known to be neuroinvasive and can cause cytokine storms, increasing nerve excitability (19, 20). It has also been suggested that children with COVID-19 may experience hypoxia, metabolic disorders, organ failure or brain damage, all of which may lead to a lower seizure threshold (21) in children with COVID-19, the underlying causes of seizure may be related to fever, encephalitis or childhood multiple system inflammatory syndrome (MIS-C), so we should be careful to diagnose febrile seizure. Considering the seriousness of children infected with COVID-19, these causes must be considered when children develop seizure.

During the follow-up, one child had new health problems, and there was no abnormality in VEEG and cerebral MRI during the follow-up. Among the reported cases, the short-term outcome of nervous system injury in most children is good, but whether there are long-term sequelae remains to be further studied. A large amount of evidence shows that the incidence of post-COVID-19 syndrome is higher in adults (22), but there are few related studies in children. Therefore, it is necessary to follow up those COVID-19 cases for a longer time and the ongoing (uncompleted data) follow up program will give us more evidence.

As a retrospective single-center study, the characteristics of COVID-19 children with febrile seizure were reported for the first time in mainland China. However, due to the low incidence of febrile seizure, the overall number of cases is small.

Based on the existing 15 cases of children, we found that the clinical manifestations of febrile seizure caused by novel coronavirus were similar to those caused by other related viruses, compared with children without febrile seizure without underlying diseases in the same age group. There was no significant difference in sex, classification, clinical manifestation and viral nucleic acid negative time, and the prognosis was good. Compared with adults, febrile seizure may be the main manifestation of COVID-19 in some children. It may occur even in children who have no history of epilepsy and are not associated with serious illness. Attention should be paid to early identification and timely improvement of the relevant nervous system examination and long-term continuous follow-up to verify the impacts on the developing nervous system.

## Data Availability

The original contributions presented in the study are included in the article/Supplementary Material, further inquiries can be directed to the corresponding author.
